# Pulmonary complications of eating disorders: a literature review

**DOI:** 10.1186/s40337-023-00735-w

**Published:** 2023-01-30

**Authors:** Allison Nitsch, Mark Kearns, Philip Mehler

**Affiliations:** 1 ACUTE Center for Eating Disorders at Denver Health, 777 Bannock Street, Denver, CO 80204 USA; 2 Department of Pulmonary and Critical Care Medicine, Denver Health, Denver, USA; 3 Department of Medicine, University of Colorado School of Medicine, 13001 E 17th Pl, Aurora, CO 80045 USA; 4 Eating Recovery Center, 7351 E Lowry Blvd, Denver, CO 80230 USA

**Keywords:** Medical complications, Pulmonary, Anorexia nervosa, Bulimia nervosa, Avoidant/restrictive food intake disorder, Binge eating disorder, Rumination, Pica

## Abstract

The medical complications of eating disorders (EDs) have been described in the literature; however, the pulmonary system has historically been described as relatively spared from the ravages of eating disorders and thus neglected in the literature. Here we review the pulmonary complications that have been described in the literature, including the effects of starvation on the lungs of nutritionally deprived animals and patients with anorexia nervosa. There is clear evidence of weakness of respiratory muscles with starvation in both groups. However, there is discordance in the literature as to whether starvation results in “nutritional emphysema,” and if so, by what mechanism and at what degree of malnutrition it develops. We also explore the growing concern for the risk of non-tuberculosis mycobacterium infection as well as risk for pneumomediastinum and pneumothorax in patients with restrictive EDs. From the limited literature, it is clear the lungs, in fact, are not spared and that further research is needed to fully understand the full extent of pulmonary complications instigated by EDs.

## Plain English summary

Eating disorders have many medical complications, however, historically the lungs have been described as spared from these complications. The authors review the available scientific literature and conclude that eating disorders do not spare the lungs as previously described and in fact may place patients at risk for weakness of the muscles that help us breath, changes to the structure of the lung, rare lung infections and potentially life-threatening collapse of the lung.

## Background

The medical complications of eating disorders (EDs) as defined by the Diagnostics and Statistical Manual for Mental Disorders (DSM-5) [1], particularly, anorexia nervosa (AN) and its subtypes, AN-restricting subtype (AN-R) and AN-binge/purge subtype (AN-BP), and bulimia nervosa (BN) have been extensively explored and reviewed in the literature [2–5]. However, historically, the pulmonary organ system has been described as “relatively spared” from the ravages of EDs. Yet, the authors have observed otherwise in their clinical practice prompting a dedicated literature review of the pulmonary complications of EDs as well as the co-occurrence of pulmonary disease and EDs.

The pulmonary organ system is made up of the lungs, a series of airways, the diaphragm, and accessory muscles of respiration which function to bring oxygen into the body and remove carbon dioxide (CO_2_) from the body. Air enters the pulmonary system through the trachea and a series of branching airways that terminate at the functional unit of the lung, the alveoli. At the level of the alveoli, oxygen crosses through the cellular wall into pulmonary capillaries where it is subsequently conducted throughout the body. Conversely, CO_2_ passes from the pulmonary circulation into the alveolus where it is exhaled. Pathology of any aspect of the respiratory system can result in clinical disease.

Clinically, respiratory function is assessed by a variety of modalities. The arterial blood gas (ABG) measures the pH of blood as well as it’s concentration of oxygen and CO_2_ to assess gas exchange. A variety of imaging modalities, the most common being x-rays and computed tomography (CT) scans, are also utilized to assess structural abnormalities of the lungs and respiratory system. Pulmonary function tests (PFTs), a non-invasive test that utilizes a variety of maneuvers to measure airflow, lung volume and capacity, gas exchange, and respiratory muscle strength, are commonly used. Of note, assessment of sleep hypoventilation, as measured through polysomnography (sleep study) or pulse oximetry (monitoring of oxygen levels in the blood) with capnography (monitoring of CO_2_ in the blood), also assesses respiratory muscle strength and is the most sensitive method to do so.

Investigations regarding the effects of eating disorders on the lungs have largely been limited to case reports and small case series. This data is further limited by variable inclusion of subtypes of eating disorders and phenotyping of disease. We hope that this comprehensive review will highlight clinical questions to focus future investigations into the effects of disordered eating and weight loss on the respiratory system.

## Methods

English language articles, case reports, and case presentations were identified for inclusion in this review utilizing PubMed and Web of Science databases. The authors searched for all eating disorders described within the DSM-5 (AN, BN, binge eating disorder (BED), avoidant/restrictive food intake disorder (ARFID), rumination disorder (RD), other specified feeding or eating disorder (OSFED), pica) in both databases combined with the following terms: pulmonary, lung function, pulmonary function, lung structure, emphysema, pulmonary structure, pulmonary infection, pneumonia, pulmonary non-tuberculosis mycobacterium, pulmonary tuberculosis, critical illness, critical care, cystic fibrosis, asthma, pulmonary fibrosis, pulmonary hypertension, pulmonary embolism.

## Animal Studies on effects of starvation on lung structure and function

In the 1970s, investigators began to explore starvation as a potential animal model of emphysema, which is a pathologic loss of alveolar tissue resulting in airspace enlargement and impaired respiratory function. Early experiments noted decreases in surfactant, increase in surface tension within the alveolar spaces and an increased propensity towards lung collapse in the lungs of starved rats compared to fed rats [6, 7]. Surfactant is a fluid produced by alveolar cells that is rich in lipids and protein. Surfactant reduces surface tension which is essential to enhance alveolar distensibility. Notably, despite reduced surfactant levels and increased propensity to alveolar collapse, the starved rats in Gail’s study did not have decreased distensibility as measured by volume-pressure curves relative to fed control rodents [7]. Starved rat lungs were also shown to have decreased protein synthesis and increased protein breakdown compared to their fed counterparts [8]. Of note, in all the aforementioned studies, the rats were only starved for 48–72 h.

Sahebjami and colleagues conducted a series of experiments on the structure and function of starved rats with the starvation period ranging from 10 to 21 days with some experiments targeting a goal reduction in body weight of 40%. Like the more time-limited investigations described above, these studies demonstrated decreased elasticity of the lung and increased surface tension in starved rodents. In addition, pathological specimens demonstrated air-space enlargement [9–11]. Electron microscopy (EM) of these samples were notable for thin and irregular alveolar walls as well as an increase in the size and number of alveolar pores (connections between alveoli) [11]. After refeeding, elasticity and surface tension returned to normal, however, air-space enlargement, while improved, did not resolve [10].

Later studies by Sahebjami’s group sought to explore the “why” of their findings on a biochemical level. They noted decreased surfactant, DNA, RNA, and protein levels in the lungs of starved rats compared to their fed counterparts [12]. The DNA, RNA, and protein levels remained significantly low after refeeding in the starved rats, while the surfactant levels normalized. This relationship led them to conclude that the amount of surfactant in the lung likely explained the changes in lung surface forces observed in prior studies. To investigate the mechanism of decreased elasticity, Sahebjami and colleagues examined connective tissue content in starved and fed rats, finding significantly lower dry and wet weights of the lung as well as lower amounts of crude connective tissue, hydroxyproline, elastin, and protein in the lungs of starved rats [13]. With refeeding, hydroxyproline content returned to normal, however, protein, crude connective tissue and elastin content only partially recovered. The authors concluded that connective tissue losses may account for some of the emphysema-like structural changes they had seen in their prior studies as well as decreased lung elasticity observed in starved rats. Sahebjami also studied the effects of starvation on young rats compared to old rats, determining that starvation in the young rats resulted in growth restriction of the lung, reduction in connective tissue, and decreased elastic forces. These changes were not observed in old, starved rats [14]. Kerr et al. had previously described similar findings of lung growth retardation in starved young rats and demonstrated emphysematous changes (reduction in alveolar surface area with increase in alveolar volume) [15]. Sahebjami et al. also demonstrated repeated “mild” starvation cycles (shorter periods of starvation than their other studies, repeated consecutively after periods of refeeding and achieving only 20% reduction in weight) in age matched young rats led to smaller total lung volumes at all pressures, reduction in hydroxyproline and crude connective tissue, and decreased alveolar surface area [16]. If the findings of these latter three can be applied to humans, they could represent significant implications for patients with restrictive EDs whose onset often occurs in adolescence during a period of rapid growth and development and can have periods of relapse and weight cycling before achieving recovery.

New rodent studies on the effects of starvation on the lung emerged in the early 2000s, containing more robust data on gene expression in the lung during the starvation period. Massaro et al. demonstrated alveolar loss as well as decreased alveolar surface area within 72 h of starvation in adult mice [17]. Microscopically, alveolar walls were thinner and alveolar space was expanded in the starved mice similar to previously performed studies. The mice were starved for a total of 15 days, with continued alveolar loss observed, however, over 75% of alveolar loss occurred within the first 72 h of starvation. The number of alveoli returned to normal with refeeding. Massaro’s study also noted gene expression and molecular changes occurring as quickly as 2 h into caloric restriction. Gene expression and molecular markers of cell death (apoptosis) and destruction of the extracellular matrix were detected. Some of the detected molecules have been associated with alveolar loss in human emphysema. In a later study, Massaro et al. gene expression that promotes tissue regeneration occurs within 1 h of resumption of diet in the starved mice [18].

Dias et al. also conducted studies on lung and diaphragm mechanics of starved rats that had lost 40% of their body weight compared to fed controls [19]. They found the starved rats had higher chest wall pressures, as well as decreased compliance, and increased resistive pressures. On histological examination, they found air space enlargement but also alveolar collapse, atelectasis, and pulmonary edema in the starved rat lungs. On EM, they identified a reduction in type II alveolar cells (cells responsible for secreting surfactant) along with structural abnormalities of cellular contents and the mitochondria of the remaining type 2 alveolar cells. EM also demonstrated respiratory muscle atrophy and collagen infiltration. The authors concluded that weakened respiratory muscles, decreased surfactant, and decreased elasticity all contributed to reduced lung function in starved rats. In a follow up study, the authors demonstrated the lung changes could only be partially reversed via nutritional supplementation [20].

From the studies of the effects of starvation on rodents, there is evidence of structural abnormalities suggestive of emphysema but also evidence of decreased lung compliance and alveolar collapse [15]. Notably, these studies reflect that the lung response is not a complete mirror of COPD or emphysema commonly associated with tobacco inhalation, where pathological changes in the airways account for airflow changes rather than abnormalities of the alveoli, leading some to refer to these findings as “nutritional emphysema [21].” Studies also support impaired respiratory muscle structure and function that impacts chest wall mechanics with starvation [19], as well as decreased lung elasticity due to reduction in elastic tissues and increased collagen. There is also evidence that surfactant production is decreased in a starved rodent which can also lead to alveolar collapse. From genetic studies, we can glean that these changes can be set into motion very rapidly in the starved rodent. The impact of starvation on lung structure and function may also be influenced by the age of the rodent at time of starvation. If this is also true in humans, findings that reduced lung growth, connective tissue content, and elasticity were only impacted in young, starved rats and not their elder counterparts, may have significant implications for patients with AN, given onset typically occurs during adolescence. These rodent findings have spurred on the study of the effects of starvation in human patients with AN.

## Human studies on effects of starvation on lung structure, function, and respiratory drive

### Historical findings

Autopsies of malnourished patients conducted by Jewish physicians in the Warsaw Ghetto during the Holocaust detailed findings of emphysema in the lungs of about 13.5% of cadavers examined the majority of whom were under 50 years old [22]. In histological specimens of cadavers observed to have emphysematous changes, 3 of the 6 were noted to have alveolar thinning and air space enlargement. The validity of these findings has been questioned by Massaro, in the context of his findings in the above-mentioned animal studies, on the basis that a standard pathological definition for emphysema was not in place at the time [21]. On living patients, the Jewish physicians also noted lowered lung borders and hyperresonance on physical exam and commensurate findings on chest x-ray (CXR) consistent with hyperinflation, a finding seen in emphysema or other decompensated airways diseases. Unfortunately, weight, height, and smoking status are not available for the cadavers or living patients in the Warsaw ghetto to objectively quantify their extent of malnutrition.

### Ventilatory function

CO_2_ plays a critical role through its impact on pH in the blood. There is a complex interconnected system of chemoreceptors in the central nervous system that drives alveolar ventilation to remove CO_2_ from the blood to tightly regulate pH. A secondary driver of respiratory effort is hypoxia (low oxygen level).

In dedicated studies of healthy adult males subjected to 10 days of starvation and “semi-starvation” conditions, the subjects were noted to have significant blunting of hypoxic respiratory drive without alteration in hypercapnia (elevated CO_2_) induced respiratory drive [23, 24]. Given the limited duration of the study, the subjects did not demonstrate significant weight loss. Conversely, in 12 patients with AN (mean body mass index (BMI) 16.1 kg/m2), González-Moro et al. demonstrated a diminished response to hypercapnia compared to healthy controls (HC). Hypercapnia was also demonstrated in a retrospective study by Kerem et al. which examined venous blood gases (VBGs) in 45 adolescent patients with AN-R (mean ideal body weight (IBW) 79%) of < 1 year duration [25]. The degree of hypercapnia correlated with their percentage of weight loss relative to IBW preceding admission. In a subsequent cohort of 16 patients, Kerem et al. redemonstrated the presence of hypercapnia and mild respiratory acidosis on admission VBGs [26]. It should be noted that VBGs may not uncover all acid- base disorders and that ABGs provide a more accurate assessment of acid–base status. Unfortunately, ABG’s were not obtained in this study. In this cohort, they also examined PFTs which were notable for decreased peak expiratory flow (PEF) but were otherwise normal relative to HCs. After weight restoration, these patients had resolution of their hypercapnia. In this cohort of patients, there were no differences noted in other markers of gas exchange, as measured by nocturnal pulse oximetry readings and end-tidal CO_2._

### Diaphragm

Normal respiratory function is influenced not only by the lung but also by the diaphragm and supporting respiratory musculature. Human study data dating back to the 1950s have demonstrated that “poorly nourished” patients without known pulmonary disease (about 71% of “normal” body weight as determined by published insurance tables from 1960) had decreased respiratory muscle strength compared to “well nourished” HCs [27, 28]. Furthermore, the diaphragm mass of “undernourished” cadavers without lung disease (again at about 71% of normal body weight based on insurance tables) has been reported to be decreased by three-fold [29].

Respiratory muscle strength can be assessed by several modalities. The most sensitive modality being sleep hypoventilation. The authors are aware of only one study that reports surrogate data for sleep hypoventilation in the form of nocturnal pulse oximetry and capnography. This study, by Kerem et al., demonstrated no significant difference between patients with AN and HC nocturnal pulse oximetry and capnography [26]. Multiple studies have demonstrated reduced maximal inspiratory (MIP) and expiratory pressures (MEP) as markers of respiratory muscle weakness in severe AN [30–32]. In the longitudinal study by Gardini Gardenghi et al., it was found that MIP and MEP decreased in the first three years of illness and then the decline appeared to plateau [30]. Of note, MIP and MEP are insensitive markers of mild respiratory muscle weakness. Respiratory muscle weakness is usually severe by the time an abnormal result is obtained. Adding further data to the previously noted PFT abnormalities, Murciano et al. also demonstrated diaphragmatic weakness in a cohort of 15 patients with AN (mean IBW of 63%) utilizing electrical phrenic nerve stimulation and maximal sniff maneuver [33]. Both measures of diaphragmatic function improved significantly 30 days into refeeding. While total lung volumes did not change between days 0 and 30 of refeeding, the vital capacity (VC), how much air is breathed out after deep inhalation, and forced expiratory volume exhaled over 1 s (FEV1), the amount of air a patient can push out of their lung in one second through forced expiration, were noted to improve significantly at 30 days. Most recently, Minano Garrido et al. measured PEF in 23 patients with AN (mBMI 11.4 kg/m^2^) [34]. They determined that these patients had decreased PEF related to depressed muscle strength which improved with weight restoration. Unfortunately, examination of full PFTs and CT imaging of the lung were not undertaken in this study, which could have provided meaningful information as this represents the study with the lowest mBMI in patients with AN to date.

### PFTs and lung imaging

While cohort studies have consistently demonstrated weakness of the diaphragm in patients with advanced AN, studies evaluating PFTs and CT imaging of the chest have been more heterogeneous. Pieters’ et al. reported PFTs in 24 patients with AN (mBMI 14.3 kg/m2) in which all PFTs were normal, including the diffusion capacity of carbon monoxide (DLCO) [32]. DLCO refers to the diffusion of carbon monoxide from the lung across the alveolar-capillary membrane into the blood stream. DLCO is used as a surrogate of the lung’s ability to exchange gases including oxygen and CO_2_. DLCO is decreased in patients with emphysema. However, it is not specific to emphysema, as DLCO can also be decreased in patients who smoke, are anemic, or have other pulmonary disease such as interstitial lung disease or pulmonary hypertension. Furthermore, DLCO can be falsely decreased in patients with respiratory muscle weakness since the measure depends on their ability to move air in and out of their lungs. The only PFT abnormalities noted included the aforementioned decreased MIP and MEP and increased residual volume (RV) which is a measurement of the volume of air remaining in the lung following maximal exhalation [32]. While no lung imaging was included in the study data, the PFT findings led the authors to presume that starvation did not induce emphysema. Of note, this study did include 9 smokers with a mean smoking history of 4.1 pack years, however, no significant difference in spirometry between smokers and non-smokers was observed.

Gardini Gardenghi et al. also studied PFTs longitudinally in patients with AN (n = 27, mBMI 16 kg/m2) [30]. In addition to the aforementioned progressive decline in MIP/MEP, they also noted significant difference in the DLCO of patients with AN compared to the HCs and standardized reference normal values. The severity of reduction in DLCO correlated with the duration of their eating disorder with a mean DLCO percent predicted of 79 ± 13% in patients with AN of less than 3 years duration and 64 ± 10% in patients with AN of greater than 3 years duration. They provided data from CT imaging obtained on this subset of patients with AN (n = 8) who had reduced DLCO on their PFTs. CT imaging of the lung utilizing lung density measurements was normal in these patients. The authors integrated their findings within the greater body of evidence to conclude that their findings support enlargement of lung alveoli without the septal destruction observed in tobacco-associated emphysema.

In contrast to Pieters et al.’s findings, Coxon et al. compared PFTs and pulmonary CTs of 21 patients with AN (mBMI 18 kg/m2) to that of HCs [35]. Four of these patients were current smokers or ex-smokers with a mean smoking history of 4 pack years. There was no significant difference in the PFT findings of patients with AN and HCs. CT images were used to calculate the volume of gas per weight of lung tissue and classified into normal lung, small emphysematous changes of lung, and large emphysematous changes of the lung based on previously validated methods in radiology literature. The patients with AN had significantly lower frequency of normal lung tissue and significantly higher frequency of small and larger emphysematous changes compared to the HCs. The authors also found that low BMI correlated with emphysematous changes on CT and predicted decreased DLCO in patients with AN. Length of illness was not found to impact CT or PFT findings. The author’s concluded that malnutrition driven emphysema-like changes occur in the lungs of patients with advanced AN.

González-Moro has also studied PFTs in patients with AN (n = 12, mBMI 16.1 kg/m2) in comparison to HCs and demonstrated a significant increase in total lung capacity (TLC), which is the volume of air within the lungs at maximal inhalation, RV/TLC ratio, and functional residual capacity (FRC), the air left in the lungs after normal, non-forced, exhalation, in patients with AN [31]. These findings are more like advanced COPD seen in tobacco smokers but were dissimilar to other AN cohorts mentioned above.

The authors could not find any studies dedicated to the examination of lung function in BN, BED, ARFID, OSFED, RD or pica. However, anecdotally, our patients that do engage in rumination as an aspect of their AN have notable bronchiectasis on lung imaging, likely secondary to repeated exposure of the lungs to gastric contents.

### Case reports

In Crow et al.’s study of mortality rate of patients with EDs, 1 of the 5 patients who had died at 10 year follow up, had emphysema listed as her cause of death [36]. Unfortunately, this 49-year-old patient’s smoking status and weight are not disclosed in the study which limits any interpretation of this finding.

Cook et al. described a case of a non-smoking 34-year-old female with AN-R at 49% IBW found to have bronchiectasis and bullae on computed tomography scan (CT) of lungs obtained during work up for progressive dyspnea on exertion and productive cough [37]. The patients' CT images were analyzed using software that predicts surface-to-volume ratios and then compared with known controlled values at their institution and were consistent with mild emphysema. The patient had PFTs which demonstrated a restrictive pattern with reduced VC and TLC. She was also noted to have reduced DLCO and reduction in maximum inspiratory and expiratory force. The patient underwent bronchoscopy and cultures obtained grew pseudomonas. Patients’ cultures were noted to be negative for mycobacterium. The authors attributed her lung disease to severe malnutrition and the case inspired further research into pulmonary complications of AN by Coxson et al. described above.

More recently, Park et al. described CT findings of large bullae, diffuse emphysema, and bronchiectasis with PFTs consistent with restrictive pattern, reduced VC, TLC, and DLCO and increased residual volume (RV) in a 43-year-old female non-smoker with AN and 47% IBW (BMI 9.8 kg/m^2^) [38]. Emphysema, bronchiectasis, and bullae as well as PFTs were noted to improve after weight restoration at 6 years. Bullous emphysema and bronchiectasis have also been reported and attributed to severe malnutrition in a 30-year-old male with AN presenting at 43% IBW [39]. Several other case reports have documented findings of emphysema and/or bronchiectasis in severely malnourished patients, each about 50% of IBW [40, 41].

Ryan et al. described respiratory dysfunction in a patient with severe AN, at 46% IBW on whom PFTs were conducted prior to and after refeeding [42]. Their patient’s TLC was 75% of predicted and improved with refeeding. This patient was also noted to have a blunted response to hypercapnia, felt to be due to respiratory muscle weakness and decreased respiratory motor neuron activity. The authors noted that patients' respiratory muscle strength was delayed in recovery compared to peripheral skeletal muscle. Another case study also highlighted delayed improvement in respiratory muscle weakness which manifested as continued difficulty with exercise tolerance despite improvement from 3.7 BMI of 15.4 to 18 kg/m^2^ in a 49-year-old male patient with AN [43].

Studies and case reports of pulmonary structure and function in AN as discussed above are summarized in Table 1. In sum, studies in malnourished patients with AN conflict in terms of PFT findings, with some studies supporting PFTs consistent with restrictive lung disease, others finding normal PFTs and a case report reflecting a more obstructive picture. It must be noted that severity of malnutrition based on mBMI or average IBW varies widely in these studies, as does average age Smokers are also clearly included in 2 of these studies, and smoking status of study participants is not clarified in 5 of these studies. Given that smoking can impact lung health and function, inclusion of smokers in these studies could confound the data presented, especially given the small study sizes. Anemia can also impact pulmonary testing. Hemoglobin is only reported in two studies, with the mean hemoglobin being normal. Pieter’s et al. do report six of their participants met criteria for anemia, all of which was mild. Given lack of reporting in most of these studies, it is unclear if anemia could be confounding study results.

**Table 1:** Summary of studies and case reports of pulmonary structure and function in anorexia nervosa

Author	Sample	Sample	Age or mean Age of AN cohort (years)	Weight	Sex	Findings
Birmingham et al.^*^ [48]	Case report	1 AN-R	49	BMI 15.4 kg/m2	M	PFTs: RMS after weight restoration to BMI 18 kg/m2. Imaging: nml CXR
Cook et al. [37]^±^	Case report	1 AN-R	34	49%IBW	F	PFTs: Restrictive pattern, marked reduction in DLCO and RMS and elevated RV. Imaging: CT c̅ bullae, bronchiectasis and mild, generalized emphysema
Coxson et al. [35]^¥^	Prospective	21 AN, 16 HC	36	mBMI 18 kg/m2	F	PFTs: As BMI decreases, DLCO decreases. No difference in RV, TLC between AN & HC. Imaging: CTs c̅ emphysematous changes
Day et al. [39] ^±^	Case report	1 AN-R	30	BMI 10 kg/m2	M	PFTs: none Imaging: CT c̅ R sided bullous disease c̅ a moderate anterior PTX, bl cavitary lesions and bronchiectasis. Dx: severe bullous emphysema
Gardini Gardenghi et al.^*^ [30]	Prospective	27 AN, 18 HC	24	mBMI 16.2 kg/m2	Not provided	PFTs: Reduced DLCO and RMS in AN vs. HC. Imaging: CT in n = 8 AN nml
Gonzalez-Moro et al. ^±^ [31]	Prospective	12 AN, 10 HC	24.7	mBMI 16.1 kg/m2	F	PFTs: Increased RV, RV/TLC, FRC and decreased RMS in AN vs HC. DLCO not undertaken. Imaging: None. Other: Reduced respiratory response to hypercapnia. No difference in ABG
Kerem et al. [25]^±^	Prospective	16 AN	15	mBMI 15.5 kg/m2	12 F, 4 M	PFTs: normal except decreased PEF. Other: Hypercapnia on admission
Kerem et al. [26]^*^	Retrospective	45 AN-R	15	mBMI 15.2 kg/m2	37 F, 8 M	Other: Mild respiratory acidosis observed in 78% of patients on admission
Minano Garrido et al.^*^ [34]	Prospective	18 AN-R, 5 AN-BP	25.6	mBMI 11.4 kg/m2	F	PFTs: Reduced RMS and PEF, improved c̅ partial weight recovery. Imaging: None
Murciano et al. [33]^*^	Prospective	15 AN	N/a	63%IBW	Not provided	PFTs: reduced RMS, reduced FEV1 and VC improved at 30 days, nml lung volumes otherwise. Imaging: None Other: normal ABGs. 4 patients with hypercapnia
Park et al. [38] ^±^	Case report	1 AN-BP	43	BMI 9.8 kg/m2	F	PFTs: Restrictive pattern; reduced VC, TLC, and DLCO. Increased RV. Improved at 6 yr f/u after 22 kg weight gain Imaging: CT c̅ large bullae of apex of L lung; diffuse emphysema and bronchiectasis. Decreased bullae size and improved emphysema at 6 yr f/u
Pieters et al. [32]^¥^	Prospective	24 AN	21.3	mBMI 14 kg/m2	F	PFTs: Reduced RMS, increased RV. Nml DLCO Imaging: None
Ryan et al. 42]^*^	Case report	1 AN-R	25	46% IBW	F	PFTs: Restrictive pattern; reduced RMS, reduced ventilatory response to hypercapnia. All improved c̅ refeeding Imaging: Nml CXR
Saran et al. [40] ^±^	Case report	1 AN-R	30	BMI 9.2 kg/m2	F	PFTs: none. Imaging: CT c̅ Bronchiectasis
Sheu et al. [41]^*^	Case report	1 AN-BP	18	BMI 10.2 kg/m2	F	PFTs: Reduced RMS. Imaging: CXR c̅ hyperinflation and decreased pulmonary vascular markings

± Non-smokers

*Smoking status not clarified

^¥^Smokers included

*ABG* Arterial blood gas, *bl* Bilateral, *DLCO* Diffusion capacity of CO_2_, *FRC* Functional reserve capacity, *PFT* Pulmonary function test, *RV* = Residual volume, *AN* Anorexia nervosa, *c̅* with, *dx* Diagnosis, *L* Left, *PTX* Pneumothorax, *TLC* Total lung capacity, *AN-BP* Anorexia nervosa, binge/purge subtype, *CT* Computed tomography of the lungs, *f/u* Follow up, *nml* Normal, *R* Right, *VC* Vital capacity, *AN-R* Anorexia nervosa, restricting subtype, *CXR* Chest x-ray, *FEV1* Forced expiratory volume in 1 s, *PEF* Peak expiratory flow, *RMS* Respiratory muscle strength, *yr* Year

These human studies seem to agree that there is diaphragmatic muscle weakness based on MIP and MEP, in some cases, resulting in hypercarbia. The studies that include DLCO are also conflicting, with some showing impaired gas exchange, which would reflect decreased alveolar surface area as seen in the above animal studies, and others reporting DLCO as normal. Lastly, the CT lung data is also conflicting with one study showing small and large emphysematous changes in the AN cohort and another demonstrating no changes, with several case reports detailing significant findings of emphysema and other structural lung changes (bullae, bronchiectasis) in patients with AN. Of note, all the case reports with emphysematous changes on CT were at significantly low IBW (less than 50%), this echoes Kerr’s animal findings where emphysema was only seen in rats that were fed one-third of their nutritional needs, and not in rats fed two-thirds of their nutritional needs for the same time period [15]. This may represent that there is a threshold effect for changes in pulmonary structure and function that is not reached until patients are at extremely low body weights. Critically, while radiographic images may demonstrate emphysematous changes, PFTs would suggest distinct physiology relative to tobacco-related emphysema which commonly results in airflow obstruction. Unfortunately, no histological or pathologic examination of the pulmonary tissues are presented in the scientific literature in starved humans with or without an ED. The authors recommend further studies that include PFTs with lung imaging be undertaken in patients IBW < 55% and compared to HCs to further elucidate the effects of starvation on pulmonary structure and function.

Given consistent evidence of a high prevalence of respiratory muscle weakness in patients with EDs, clinicians should be cognizant of potentially impaired reserve and risk of progression to respiratory failure in patients with ED. Early symptoms of respiratory failure can include fatigue, sleepiness, and/or headache while later symptoms include tachypnea, tachycardia, lethargy, and/or subtle change in oxygenation. Any of these symptoms should warrant evaluation with pulmonary imaging studies and a low threshold for obtaining ABG analysis to facilitate early identification and treatment of respiratory disease. Lastly, the authors would not recommend the use of VBGs to assess acid–base status in patients with EDs given their risk for mixed acid–base disorders, with both respiratory and metabolic compensation, which can only be adequately assessed via ABG.

## Pulmonary infections and eating disorders

Despite severe protein calorie malnutrition being a risk factor for infection in patients without restrictive eating disorders, malnourished patients with AN do not appear to share this risk [44]. In fact, patients with malnutrition from AN, seem to be protected from viral infections[45] and bacterial infections [44]. Severe protein calorie malnutrition in patients without AN is marked by changes in humoral and cellular immunity. In contrast, alterations in humoral immunity are not seen in patients with AN, however, alterations in cellular immunity have been described [46]. Specifically, CD8 + T cell populations are decreased, which increases the CD4 + /CD8 + Ratio, and leads to decrease in memory CD8 cells [46]. CD8 + T cells are involved in viral defense and production of interferon gamma (IFN-gamma). These changes resolve with refeeding. Other T cell populations do not appear to be altered in malnourished patients with AN. Studies have demonstrated changes in neutrophil's bactericidal ability in patients with AN, but this does not seem to increase their risk for infection [46].

### Bacterial pneumonia

In a comprehensive review, Brown et al. reviewed 311 consecutive admissions of patients with AN to their unit and looked at occurrence of infection during hospital course [47]. Twenty-three patients were found to have bacterial infection during their admission, 5 of which were pneumonia. This made pneumonia the most common bacterial infection observed in this cohort of patients with AN, accounting for 19% of infectious cases. Notably, there was no significant difference between recorded body temperatures of AN patients with infection and patients with AN without infection. Most patients with serious infection demonstrated no fever during their infectious course. Patients with AN and infection were then matched and compared with age and type of infection matched controls. The patients with AN were less likely to demonstrate a fever than their non-AN counterparts and were more likely to develop secondary complications from their infection. Birmingham et al. also presented 5 cases of infection requiring hospitalization in patients with AN, two of which were for pneumonia [48]. None of the patients had temperatures greater than 37 °C during their disease course.

Anecdotally, bacterial pneumonia is the most common type of infection encountered by the authors in their clinical practice which encompasses hospitalized patients with severe malnutrition from AN-R, AN-BP or ARFID (typically less than 70% IBW). The pneumonia in the authors clinical practice is most commonly due to aspiration, even in the absence of self-induced vomiting, due to oropharyngeal muscle weakness. The authors reviewed the literature on pulmonary infections and eating disorders. Unfortunately, the authors could not find any studies dedicated to pulmonary infection occurrence in BN, BED, ARFID, or pica. Also in the authors’ clinical practice, the patients with severe malnutrition and pneumonia respond to standard antibiotic regimens.

### Mycobacterial and other infections

An emerging area of concern is the incidence of non-tuberculosis mycobacteria (NTM) pulmonary infection in patients with AN. NTM are ubiquitously found in the environment, primarily in wet soil. Previously, NTM has been associated with diverse patient populations including structural lung disease, such as COPD, bronchiectasis, and cystic fibrosis, along with post-menopausal women and patients with human immunodeficiency virus infection and acquired immunodeficiency syndrome (HIV/AIDS) [49]. The authors found 10 case reports of patients with AN and one case report of a patient with ARFID who were found to have NTM [50–59], the majority of which were published in the last 10 years. Thin body habitus has been postulated to be a risk factor for NTM due to decreased leptin levels and the role leptin plays in host immune defense, specifically, its role in T cell differentiation towards T cells that produce IFN-gamma [49]. Host defense against tuberculosis and NTM relies on IFN-gamma through activation of macrophages and granuloma formation [60]. Patients with AN and BN also have been shown to have decreased leptin levels [61], which could possibly explain the occurrence of NTM in patients with AN and BN. Reflux and aspiration have also been noted to be risk factors for NTM. As self-induced vomiting is associated with reflux and aspiration, one could hypothesize that patients with AN-BP would be at increased risk. However, only 1 of the case reports is clearly identified as AN-BP, 2 are identified as AN-R, and the remainder are generically labeled AN. Also of note, WBC was reported in 8 of the 11 case studies and in all but 1 of the cases where the information is reported, WBC was normal or elevated, in contrast to the leukopenia (low white blood cell count) typically associated with severe malnutrition in restrictive EDs [62]. Furthermore, female patients with severe malnutrition also have low levels of estrogen, like post-menopausal women, which is also felt to play a role in immune defense and may play a role in the pathogenesis of disease. This is an important area for further investigation as Pulmonary NTM treatment is complex requiring extended treatment courses of multiple antibiotics and occasionally surgical resection of the infected lung. Nutritional status in and of itself can be a predictor of mortality in NTM [63], with patients without severe eating disorders requiring intense nutritional support. In the author’s experience patients with pulmonary NTM and severe malnutrition due to an ED have higher caloric requirements than similarly weighted peers seeking nutritional rehabilitation.

Reports of pulmonary tuberculosis in patients with AN have also been reported in the literature [64–66], though are far less prevalent than NTM owing to its rarity in the general population of the United States. Of note, in these reports, all patients had normal or elevated WBC. Invasive pulmonary aspergilloma has also been reported in patients with AN [67–69], one of which was fatal. The two non-fatal cases were managed surgically with good response. These patients again were described as having normal WBC. Lastly, a case of *P. jirovecci* pneumonia (PCP) in a patient with severe malnutrition from AN has been described in the literature. The patient developed respiratory failure during hospital admission for refeeding and no other risk factors such as immunosuppression from chemotherapy, chronic steroid use, or HIV/AIDS were present. The patient was noted to have leukopenia and a low CD4 count.

In sum, the incidence of bacterial pneumonia in patients with AN compared to the general population is unclear. Patients with AN may be at risk for pulmonary NTM due to several different biochemical or mechanical factors compared to the general population. Physicians must be vigilant in monitoring for infection in patients with AN as they are unlikely to display fever. Furthermore, in severe malnutrition from AN, a low WBC count is “normal,” and a normal or elevated WBC count should raise suspicion for covert infection even in the absence of focal symptoms.

## Pneumomediastinum and pneumothorax and eating disorders

The authors have previously reviewed the occurrence of pneumothorax (PTX) and pneumomediastinum (PM) in restrictive EDs (AN-R, AN-BP, ARFID) at their institution and in the scientific literature [70]. The authors could not identify literature describing PM and/or PTX in BED, OSFED, RD or pica. Spontaneous PM, air trapped in the interstitium of the thoracic cavity, does occur with some frequency in patients with AN-R and AN-BP, though its occurrence in this population compared to the general population is unclear [70, 71]. While we have hypothesized that the increased intrathoracic pressure generated by self-induced vomiting would increase rates of PM in patients with AN-BP, spontaneous PM is equally reported in AN-R and AN-BP. Typically, the findings of spontaneous PM were incidentally noted on imaging obtained for other purposes. There is a well-described phenomenon resulting in PM, called the Macklin effect, in which air tracks from a ruptured alveolus along the bronchovascular bundle back to the mediastinum resulting in PM [72]. If the alveolar thinning noted in animal studies correlates to humans with eating disorders, these patients may be at increased risk for PM relative to patients without eating disorders related to the Macklin effect. After secondary causes for PM are ruled out, such as esophageal rupture, PM can be managed conservatively without intervention.

Pneumothorax, trapping of air between the chest wall and the lung, has been reported in 24 case reports in the literature in patients with AN-R, AN-BP, and ARFID [70]. Pneumothorax can be classified as spontaneous, traumatic, or iatrogenic. Spontaneous PTX can be classified as primary or secondary (due to underlying chronic lung disease). From the available case studies, it appears that spontaneous and iatrogenic PTX are equally common in the restrictive ED population (accounted for 10 cases each). Iatrogenic PTX occurred due to pacemaker placement, central line insertion, thoracentesis, nasogastric tube placement, chest tube placement, bronchoscopy, and abdominal surgery. Spontaneous pneumothorax in this population may be related to the development of blebs or bullae caused by malnutrition, given that these structural abnormalities are thought to be risk factors for spontaneous PTX in the general population [73, 74]. The traumatic PTXs presented in the literature could be directly attributed to the patients’ EDs as they were results of falls or cardiac arrest due to severe electrolyte disturbances related to the patients’ underlying ED.

Given known occurrence of PM and spontaneous PTX in patients with restrictive EDs, the authors recommend CXR be pursued in patients with any chest or respiratory symptoms. Furthermore, extreme caution is recommended when performing procedures overlying the thoracic cavity, such as pacemaker placement, thoracentesis, and central line insertion in this patient population. When the potential benefit of invasive procedure exceeds this increased risk, thoracentesis and central line insertion should only be completed on these patients by extremely experienced physicians under ultrasound guidance.

## Asthma and eating disorders

Asthma is a pulmonary disease in which the airways narrow due to inflammation or known triggers resulting in temporary airflow obstruction resulting in wheezing and respiratory distress. The Jewish physicians in the Warsaw ghetto anecdotally reported a decrease in asthma exacerbations in their malnourished patients with asthma [22]. The authors could not identify any studies that corroborate their reports in modern scientific literature on malnourished patients with EDs. Aulinas et al. have found that asthma is more prevalent in patients with ARFID than in AN [75]. Asthma prevalence in patients with ARFID was noted to be 25% in this study, whereas it was reported to be 2% in AN and 8% in HCs. Another study by Ghadirian et al. demonstrated a life-time prevalence of asthma to be 10% in patients with AN compared to 8.7% in patients with BN [76]. This study unfortunately did not include HCs, however, the prevalence of asthma in the United States has been reported to be around 8% [77]. In a separate study, asthma was not found to be associated with greater risk of total EDs, however, it has been noted to be significantly higher odds of BED and lower odds of BN [78]. RD has been found to have an association with asthma in patients that also have functional dyspepsia [79]. Chronic illness, such as asthma, has been associated with high-risk weight-loss practices in adolescents [80]. From the available literature, it is unclear the impact eating disorders have on asthma, however, having asthma may increase a patient’s risk for developing an eating disorder.

## Cystic fibrosis and eating disorders

Cystic fibrosis (CF) is a genetic disease which, through production of abnormal mucous, causes inflammation in and damage to the lungs, and increases the afflicted patient’s risk of pulmonary infection. Also due to the implications of the genetic mutation, patients with CF are at risk for malnutrition and the complications that accompany it. Obviously, given its genetic nature, EDs cannot cause CF. Adequate nutrition is a focus of CF treatment, as malnutrition can further complicate the pulmonary disease course. Gilcrest et al. have suggested that this preoccupation with weight can predispose patients to disordered eating and the subsequent development of an ED [81]. Considerable focus has been given to disordered eating and EDs and the potential prevalence of their co-occurrence in the CF literature [82]. With Petropoulou et al. concluding, “Disordered eating behaviors and EDs appear common in CF, although it is unclear if the prevalence is greater or similar to that of the general population.” Providers who care for patients with CF should therefore maintain a high index of suspicion for EDs in their patient population, especially given the clinical implications of malnutrition on their disease course.

## Pulmonary fibrosis and pica

There has been a single case report denoting development of pulmonary talcosis in a patient with pica which drove her to consume talcum powder orally for over 20 years [83]. The patient presented with hemoptysis and was found on CT scan to have bilateral apical consolidative airspace opacities with adjacent branching nodular densities and ground-glass opacification with innumerable miliary nodules which were found on lung biopsy to be consistent with pulmonary talcosis. Therefore, potential pulmonary toxicity of the “non-food item” of choice should be considered in patients with pica and prompt swift intervention to prevent chronic lung damage.

## Limitations and conclusions

This review is limited by lack of literature available on the impact of the BN, ARFID, BED, OSFED, RD, and pica on the lungs. It is unclear if this lack of literature is due to lack of pulmonary complications associated with these EDs or due to research on medical complications of EDs being historically focused on AN, despite BED being the most common ED in the United States [84]. The limited literature on ARFID may be due to its relatively new classification as an ED in the DSM-5.

The authors conclude that malnutrition in AN results in respiratory muscle weakness and this should be considered in patient management during episodes of respiratory distress. Malnutrition may result in emphysematous changes on lung imaging in patients with extreme AN. Animal studies suggest that the mechanism for these emphysematous changes is due to a decrease in surfactant, elastin, and cell signaling pathways that may increase apoptosis brought about by the starved state. Further studies are needed on patients with severe malnutrition from their AN compared to HC at lower body weights looking at PFTs and lung imaging to further determine the occurrence of emphysema-like changes that may occur in this population given concern for a threshold effect for these findings. Moreover, future studies should compare young and old patients. Care should also be taken in future studies to avoid confounders such as smoking status and anemia.

Furthermore, in patients with AN, a high index of suspicion must be maintained even in the absence of fever for pneumonia or other bacterial infections, especially in the setting of laboratory findings of normal or elevated WBCs. NTM in AN may be an emerging pathogen of concern. Patients with recurrent pneumonia or structural lung changes such as bronchiectasis should be evaluated for NTM. The authors also recommend that patients with malnutrition presenting for care with complaint of chest pain, shortness of breath, or other vague respiratory/chest complaints be evaluated with CXR to evaluate for PTX or PM. Lastly, we recommend that risk of procedures in or around the chest cavity such as thoracentesis or central line placement be considerably weighed against potential benefit given risk of iatrogenic PTX in malnourished patients with AN.

## Data Availability

Not applicable.

## Abbreviations

ABG: Arterial blood gas
AN: Anorexia nervosa
AN-R: Anorexia nervosa, restricting subtype
AN-BP: Anorexia nervosa, binge/purge subtype
ARFID: Avoidant/restrictive food intake disorder
BN: Bulimia nervosa
BED: Binge Eating disorder
CF: Cystic fibrosis
CO_2_: Carbon dioxide
CT: Computed tomography scan
CXR: Chest X-ray
DLCO: Diffusion of carbon monoxide
ED: Eating disorder
EM: Electron microscopy
FEV1: Forced expiratory volume in one second
FRC: Functional residual capacity
HC: Healthy controls
HIV/AIDS: Human immunodeficiency virus infection and acquired immunodeficiency syndrome
IBW: Ideal body weight
mBMI: Mean body mass index
MEP: Maximal expiratory pressure
MIP: Maximal inspiratory pressure
OSFED: Other specified feeding or eating disorder
NTM: Non-tuberculosis mycobacterium
PCP: *P. jirovecci* Pneumonia
PEF: Peak expiratory flow
PFT: Pulmonary function tests
PM: Pneumomediastinum
PTX: Pneumothorax
RD: Rumination disorder
RV: Residual volume
TLC: Total lung capacity
VBG: Venous blood gas
VC: Vital capacity
WBC: White blood cell count
